# Machine Learning Empowering Drug Discovery: Applications, Opportunities and Challenges

**DOI:** 10.3390/molecules29040903

**Published:** 2024-02-18

**Authors:** Xin Qi, Yuanchun Zhao, Zhuang Qi, Siyu Hou, Jiajia Chen

**Affiliations:** 1 School of Chemistry and Life Sciences, Suzhou University of Science and Technology, Suzhou 215011, China; 2111121006@post.usts.edu.cn (Y.Z.); 2211121003@post.usts.edu.cn (S.H.); njucjj@126.com (J.C.); 2 School of Software, Shandong University, Jinan 250101, China; z_qi@mail.sdu.edu.cn

**Keywords:** machine learning, drug discovery, transformer, opportunity, challenge

## Abstract

Drug discovery plays a critical role in advancing human health by developing new medications and treatments to combat diseases. How to accelerate the pace and reduce the costs of new drug discovery has long been a key concern for the pharmaceutical industry. Fortunately, by leveraging advanced algorithms, computational power and biological big data, artificial intelligence (AI) technology, especially machine learning (ML), holds the promise of making the hunt for new drugs more efficient. Recently, the Transformer-based models that have achieved revolutionary breakthroughs in natural language processing have sparked a new era of their applications in drug discovery. Herein, we introduce the latest applications of ML in drug discovery, highlight the potential of advanced Transformer-based ML models, and discuss the future prospects and challenges in the field.

## 1. Introduction

Drug research and development play a vital role in improving human health and well-being. However, the discovery of a new drug is an extremely complex, expensive and time-consuming process, typically costing approximately USD 2.6 billion [1] and taking more than 10 years on average [2]. Despite the high investment levels, the approval success rate of launching a small-molecule drug to market from phase I clinical trial is less than 10% [3], highlighting the considerable risk of failure. Therefore, how to reduce the costs and accelerate the pace of new drug discovery has emerged as a key concern within the pharmaceutical industry.

**Figure 1:**
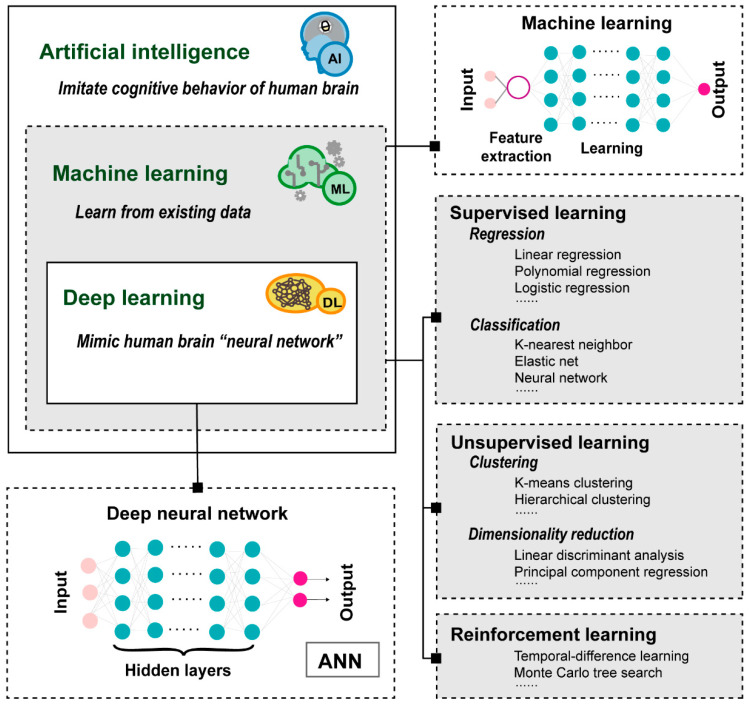
Introduction diagram of artificial intelligence and its subfields: machine learning and deep learning.

The increasing availability of large-scale biomedical data provides tremendous opportunities for computational drug discovery, but effectively mining, correlating, and analyzing these huge amounts of data has become a critical challenge. Fortunately, with the advent of efficient mathematical tools and abundant computational resources, artificial intelligence (AI) approaches have rapidly developed (Figure 1). As the representative AI method, machine learning (ML), empowers machines to learn from existing data by using statistical approaches and make predictions, which can be further classified into supervised, unsupervised, and reinforcement learnings [4,5]. Deep learning (DL), a subset of ML, focuses on using multi-layered artificial neural networks (ANNs) structures to simulate the neural networks of the human brain for learning data representations, making it more powerful and flexible in handling complex and high-dimensional data [6,7]. With the advantages of low cost and fast speed, the ML approaches are revolutionizing and strengthening multiple stages of drug discovery, such as target identification, *de novo* drug design and drug repurposing. For example, DL-based open-source tools, such as DeepDTAF [8] and DeepAffinity [9], have been applied to predict the binding affinity of drug–target interactions (DTIs), making the hunt for new pharmaceuticals more efficient. Accordingly, more and more pharmaceutical giants, such as Sanofi (Paris, France), Merck (Darmstadt, Germany), Takeda (Takeda, Japan) and Genentech (South San Francisco, America), have initiated cooperation with AI companies to develop new drugs.

Notably, the Transformer-based language models, such as the Generative Pre-training Transformer (GPT), Bidirectional Encoder Representations from Transformers (BERT) and the Text-to-Text Transfer Transformer (T5), have not only achieved revolutionary breakthroughs but have also brought about a paradigm shift in the area of natural language processing (NLP) [10]. In particular, the outstanding learning ability, generalization ability and transferability of Transformer-based language models have sparked a new era of their applications in drug discovery and development, primarily owing to the inherent similarities between drug-related biological sequences and natural languages. Their remarkable advantages, including capturing long-range dependencies in sequences, processing input sequences in parallel, employing an attention mechanism, and having extendibility to incorporate multimodal information, make them valuable tools for various aspects of the drug discovery process [11]. For example, by employing Transformer-based language models, Kalakoti et al. [12] have successfully developed a modular framework called TransDTI for predicting novel DTIs from sequence data. Its performance proved to be superior to existing methods. Therefore, the Transformer-based models have the potential to revolutionize the identification and development of new drugs.

Given the significance of ML techniques in the pharmaceutical industry, we here focus on introducing the recent advancements, opportunities and challenges of ML applications in drug discovery. First, we provide an updated overview of the emerging applications of ML in different stages of the drug discovery process, including drug design, drug screening, drug repurposing and chemical synthesis. Next, we highlight the opportunities of the advanced Transformer-based models in empowering drug discovery. Furthermore, we discuss the challenges and future prospects of ML in the field of drug discovery.

## 2. Applications of ML in Drug Discovery

**Figure 2:**
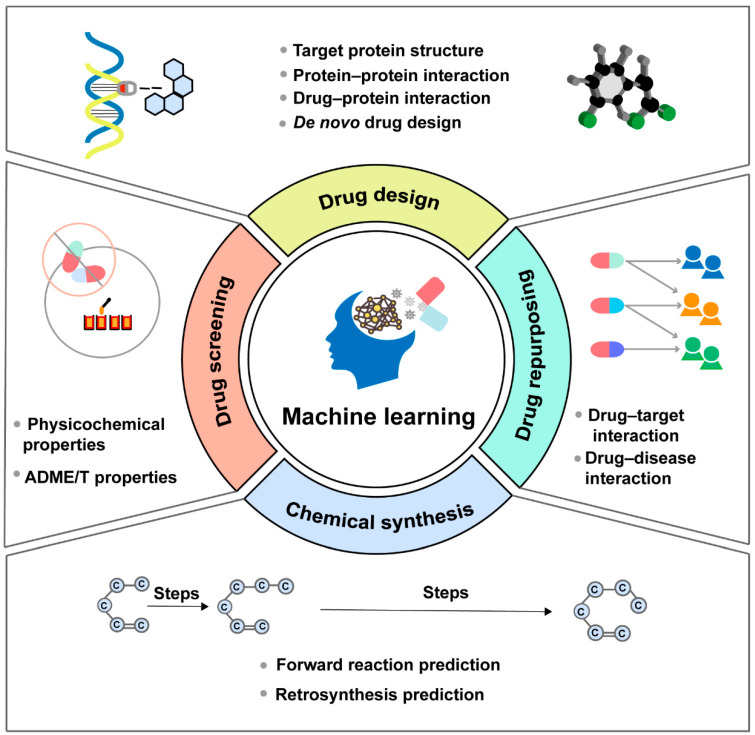
Machine learning can be applied in multiple stages of the drug discovery process, mainly including drug design, drug screening, drug repurposing and chemical synthesis.

The process of discovering effective new drugs is time-consuming and predominantly the most challenging part of drug development. With the advantages in learning from data, discerning patterns, and making intelligent decisions, ML-based approaches have emerged as versatile tools that can be applied in multiple stages of drug discovery, including drug design, drug screening, drug repurposing and chemical synthesis (Figure 2). Moreover, considerable efforts are dedicated to developing models, tools, software and databases based on the core architecture of ML algorithms, to counter the inefficiencies and uncertainties inherent in traditional drug development methods (Table 1).

### 2.1. Application of ML in Drug Design

#### 2.1.1. Prediction of the Target Protein Structure

Since proteins play crucial roles in various biological processes, their dysfunctions can lead to abnormal cell behavior and lead to the development of diseases [86]. For selective targeting of diseases, small-molecule compounds are generally designed based on the three-dimensional (3D) chemical environment surrounding the ligand-binding sites of the target protein [87]. Hence, predicting the 3D structure of the target protein is of great significance for structure-based drug discovery. Homology modeling has traditionally been used for this purpose, relying on known protein structures as templates [88]. Comparatively, ML-based approaches have shown great promise in predicting the 3D structures of target proteins with improved accuracy and efficiency. For example, AlphaFold is a state-of-the-art protein structure prediction system developed by DeepMind, a leading AI company. Based on deep neural network (DNN), it has achieved remarkable success in multiple protein structure prediction competitions, demonstrating its ability to accurately predict the 3D structures of proteins by analyzing the adjacent amino acid distances and peptide bond angles [14]. Importantly, AlphaFold has significantly advanced the field of protein structure prediction and has the potential to revolutionize drug discovery [14]. Therefore, ML-based approaches hold great potential to enhance our understanding of protein structures. It should be noted that protein structures can undergo changes in different environments, and proteins may form multiple coexisting structures under the same conditions [89]. This complexity adds to the challenges of structure prediction.

#### 2.1.2. Prediction of PPIs

In most cases, proteins rarely implement their functions alone, but rather cooperate with other proteins to form intricate relationships known as the protein–protein interaction (PPI) network [86]. PPIs possess indispensable functions in diverse biological processes. They can contribute to altering protein specificity, regulating protein activity and generating novel binding sites for effector molecules [90]. Hence, understanding and targeting PPIs offers opportunities to design innovative drugs that can modulate complex biological processes.

Currently, ML-based methods for PPI prediction can be broadly grouped into structure-based and sequence-based categories. Structure-based approaches mainly leverage the knowledge of protein structure similarity to predict PPIs [91]. For example, IntPred, a random forest ML tool, was developed to predict protein–protein interface sites based on structural features. Compared with other methods, the IntPred predictor showed strong performance in identifying interactions at both the surface-patch and residue levels on independent test sets of both obligate and transient complexes (Matthews’ Correlation Coefficient (MCC) = 0.370, accuracy = 0.811, specificity = 0.916, sensitivity = 0.411) [18]. Struct2Graph, a graph attention network (GAT)-based classifier, was proposed to identify PPIs directly from the 3D structure of protein chains [24]. The accuracy of Struct2Graph on balanced sets with equal numbers of positive and negative pairs was 0.9989, and the average accuracy of five-fold cross-validation on unbalanced sets with a ratio of positive and negative pairs of 1:10 was 0.9942 [24]. Comparatively, sequence-based PPI prediction approaches aim to identify physical interactions between two proteins by leveraging information from their protein sequences [92]. DNNs provide a robust solution for this purpose. They are composed of multiple layers of interconnected neurons, allowing them to automatically extract complex patterns and features from data. For example, DeepPPI applied DNNs to effectively learn protein representations from common protein descriptors, thereby contributing to the prediction of PPIs. It can achieve excellent performance on the *S. cerevisiae* dataset with an accuracy of 0.925, precision of 0.9438, recall of 0.9056, specificity of 0.9449, MCC of 0.8508 and area under the curve (AUC) of 0.9743, respectively [27]. Extensive experiments showed that DeepPPI was able to learn the useful features of protein pairs through a layer-wise abstraction, resulting in better predictive performance than existing methods on core *S. cerevisiae*, *H. pylori* and *H. sapien* datasets [27]. In addition, based on Uniprot database, Li et al. [20] developed a DELPHI, a new sequence-based deep ensemble model for PPI-binding sites’ prediction. Therefore, ML-based approaches have great potential in enhancing the identification of PPI sites. Compared with sequence-based approaches, structure-based ones are limited by the scarcity of available protein structures and the low quality of familiar protein structures [90,93].

#### 2.1.3. Prediction of DTIs

Most drugs exert therapeutic effects by specifically interacting with target molecules within the body, such as enzymes, receptors and ion channels. Hence, the accurate prediction of DTIs is a pivotal step in the drug design pipeline. As the traditional experimental approaches are time-consuming and costly, ML-based methods have been increasingly developed and applied by researchers to predict DTIs. These methods primarily focus on three key aspects: predicting the binding sites of drugs on target molecules, estimating the binding affinity between drugs and targets, and determining the binding pose or conformation of the drug within the target molecule [94].

Firstly, binding sites, also referred to as binding pockets, are specific locations within a protein where interactions occur between the protein and a ligand (such as a drug molecule) [94]. By introducing a deep convolutional neural network (CNN), Cui et al. [32] developed a sequence-based method, DeepC-SeqSite, for predicting protein–ligand binding residues. Notably, this method exhibited superior performance compared with multiple existing sequence-based and 3D-structure-based methods, including the leading ligand-binding method COACH [32]. Similarly, Zhou et al. [36] proposed a binding site prediction method called AGAT-PPIS based on augmented GAT. It demonstrated significant improvements over the state-of-the-art method, achieving an accuracy increase of 8% on the benchmark test set. Moreover, binding affinity represents the strength of an interaction between a drug and its target. Some tools based on ML and DL algorithms have been applied to determine DTIs’ binding affinity, such as DEELIG [39] and GraphDelta [40]. In addition, the active conformation of ligands plays a crucial role in facilitating the effective binding between proteins and drugs [94]. By combining random forest and CNN strategies, Nguyen et al. [44] proposed a scoring function to select the most relevant poses generated by docking software tools including GOLD, GLIDE and Autodock Vina, thereby contributing to obtaining more accurate and effective ligand–target binding configurations. Therefore, ML algorithms have been extensively employed to predict DTIs and hold the potential to facilitate the design of new drugs.

#### 2.1.4. *De Novo* Drug Design

*De novo* drug design refers to the process of creating new drug molecules from scratch using computational methods, without relying on existing bioactive compounds or known drug structures. It involves designing molecules that have specific properties and functions to target a particular disease or condition [95,96]. Compounds developed with traditional *de novo* drug design methods (e.g., the fragment-based approach) usually have poor drug metabolism and pharmacokinetics properties and are hard to synthesize due to the complexity and impracticality of compound structures [97,98]. Therefore, there is high demand for new methods to explore chemical entities that meet the requirements of biological activity, drug metabolism, pharmacokinetics and synthesis practicality.

Recently, ML-based approaches, especially auto-encoder variants (e.g., the variational auto-encoder (VAE) and adversarial auto-encoder (AAE)) have gained attention in the field of *de novo* drug design. PaccMann^RL^ is an example of these approaches that combines a hybrid VAE with reinforcement learning for the *de novo* design of anti-cancer molecules from transcriptomic data [49]. Similarly, another approach, known as druGAN, utilizes a deep generative AAE model to generate novel molecules that possess specific anti-cancer properties [50]. In addition, a Wasserstein GAN and GCN-based model, known as MedGAN, has been successfully developed to generate novel quinoline-scaffold molecules from complicated molecular graphs and evaluate drug-related properties [56]. It has been demonstrated that the MedGAN was able to produce 25% effective molecules, 62% fully connected, among which 92% are quinoline, 93% are novel, and 95% are unique [56]. To address the difficulty in synthesizing generated molecules, Coley et al. [51] defined a synthetic complexity score, namely SCScore, that utilizes precedent reaction knowledge to train a neural network model for evaluating the level of synthetic complexity. Therefore, ML-empowering approaches play crucial roles in *de novo* drug design, revolutionizing the process of discovering and developing new drugs.

### 2.2. Application of ML in Drug Screening

#### 2.2.1. Prediction of the Physicochemical Properties

The physicochemical properties of drugs, mainly including solubility, ionization degree, partition coefficient, permeability coefficient and stability, play a significant role in determining their behavior (e.g., bioavailability, absorption, transportation and permeability) in biological systems as well as the environment, and in evaluating their potential risks to human health [6,59]. Hence, these properties are assessed during drug screening to select promising candidates for further development and optimization. At present, multiple ML-based tools have been proposed to predict the physicochemical properties of molecules. For example, Francoeur et al. [58] developed a molecule attention Transformer called SolTranNet for predicting aqueous solubility from the SMILES representation of drug molecules. It has been demonstrated to function as a classifier for filtering insoluble compounds, achieving a sensitivity of 0.948 on Challenge to Predict Aqueous Solubility (SC2) datasets, which is competitive with other methods [58]. Moreover, by using molecular fingerprints and four ML algorithms, Zang et al. [59] developed a quantitative structure–property relationship workflow to predict six physicochemical properties of environmental chemicals, such as water solubility, octanol–water partition coefficient, melting point, boiling point, bioconcentration factor, and vapor pressure [59]. Therefore, these ML-based predictors are valuable tools in drug discovery, as they can help in screening potential drug candidates based on their physicochemical properties.

#### 2.2.2. Prediction of the ADME/T Properties

Once hit or lead compounds are identified during the drug discovery process, a series of tests and evaluations are conducted to assess their absorption, distribution, metabolism, and excretion and toxicity (ADME/T) properties [99]. These pharmacokinetic properties are essential for understanding how the compounds will behave in the human body and whether they have the potential to be safe and effective as drugs. Imbalanced ADME/T properties frequently cause the failure of drug candidates in late stages of drug development and may even lead to the withdrawal of approved drugs [100]. Hence, ADME/T properties are often employed as molecular filters to screen large databases of compounds in the early stage of drug discovery, thereby helping to increase efficiency and improve the success rate of drug screening [93,100].

To detect the ADME/T properties of drugs, various evaluation criteria such as hepatotoxicity, passing through the blood–brain barrier (BBB), plasma protein binding (PPB) and cytochrome P450 2D6 (CYP2D6) inhibition are commonly used [101,102]. Accordingly, there has been growing interest in developing ML-based tools for the prediction of these criteria. For example, Tian et al. [60] developed a web server called ADMETboost that utilizes the powerful extreme gradient boosting (XGBoost) model to learn about molecule features from multiple fingerprints and descriptors, allowing for the accurate prediction of ADME/T properties, such as Caco2, BBB, CYP2C9 inhibition, CL-Hepa and hERG. It has been demonstrated that this model can achieve remarkable results in the Therapeutics Data Commons ADMET benchmark, ranking first in 18 out of 22 tasks and within the top three in 21 tasks [60]. Similarly, by utilizing more than 13 000 compounds obtained from the PubChem BioAssay Database, Li et al. [65] proposed a multitask autoencoder DNN model to predict the inhibitors of five major cytochrome P450 (CYP450) isoforms (1A2, 2C9, 2C19, 2D6 and 3A4). Especially, the multi-task DNN model achieved average prediction accuracies of 86.4% in 10-fold cross-validation and 88.7% on external test datasets, outperforming single-task models, earlier described classifiers and conventional ML methods [65]. Furthermore, the Tox21 Challenge is a collaborative effort aimed at developing predictive models for toxicity assessment using high-throughput screening data. In this context, Mayr et al. [64] developed a DL pipeline, DeepTox, for toxicity prediction. It outperformed all other computational methods (e.g., naïve Bayes, random forest and support vector machine) in 10 out of 15 cases in the Tox21 Challenge [64]. Therefore, ML algorithms have made significant progress in predicting the ADME/T properties of drugs, contributing to guiding drug safety assessment and preclinical research.

### 2.3. Application of ML in Drug Repurposing

Drug repurposing, also known as drug repositioning, is a strategy to identify new indications from approved or investigational (including failed in clinical trials) drugs that have not been approved [103]. As this approach takes advantage of the extensive safety testing conducted during clinical trials for other purposes, repurposing known drugs not only speeds up the drug development process but also presents cost-saving advantages compared to developing entirely new drugs from scratch [103]. Currently, researchers are increasingly developing and applying ML-based methods to conduct drug repurposing, which can be broadly divided into target-centered and disease-centered approaches [104].

In target-centered drug repurposing, network-based methods have been widely applied to search new targets for known drugs. For example, by employing autoencoder and Positive-Unlabeled matrix completion algorithms, Zeng et al. [70] developed a calculation method called deepDTnet to identify new targets for known drugs from a heterogeneous drug–gene–disease network. Experiments have shown that the deepDTnet achieved a high accuracy in predicting new targets of existing drugs (AUC = 0.963), which is superior to traditional ML methods [70]. Similarly, by combining the network diffusion algorithm and the dimensionality reduction approach, Luo et al. [72] developed DTINet, a novel network-integration procedure for DTI prediction and drug repositioning. It can outperform other existing methods, with AUC and area under precision-recall (AUPR) 5.7% and 5.9% higher than the second best method, respectively, providing an effective tool in the field of drug discovery and target identification [72].

In addition, disease-centered approaches are mainly aimed at identifying drug–disease relationships and can be widely divided into similarity-based and network-based ones [104]. Similarity-based methods have achieved significant progress by combining drug or disease characteristics with the known drug–disease associations [104]. For example, based on the assumption that similar drugs are commonly associated with similar diseases, Luo et al. [73] proposed a novel computational approach called MBiRW, which combines similarity measurements and a Bi-Random walk algorithm to recognize potential novel indications for a specific drug. MBiRW can achieve a high accuracy in predicting drug–disease associations (AUC = 0.917), which is superior to other methods [73]. In addition, network-based methods integrate information from different biological networks to improve the predictive accuracy of drug–disease relationships. For example, Doshi et al. [74] developed a graph neural network model called GDRnet for drug repurposing, which can efficiently screen existing drugs in the database and predict their unknown therapeutic effects by evaluating the scores of drug–disease pairs. Therefore, ML technology holds significant promise in the field of drug repurposing, providing strong support for accelerating drug discovery.

### 2.4. Application of ML in Chemical Synthesis

Organic synthesis is a key part of the small-molecule drug-discovery process [97]. New molecules are synthesized along the path of compound optimization to achieve improved properties. To promote molecule synthesis, researchers have developed multiple ML-based computational tools applicable to the retrosynthesis prediction and forward reaction prediction.

#### 2.4.1. Retrosynthesis Prediction

Retrosynthesis planning aims to identify efficient synthetic routes for a desired molecule by recursively converting it into easier precursors. Therefore, it can effectively solve the synthesis of complex molecules to facilitate the development of organic synthesis science [105]. At present, a series of ML-based approaches have been used for retrosynthesis planning, mainly including template-based and template-free approaches.

The template-based approach involves systematically comparing the target molecule with a set of templates, each representing alternative substructure patterns that occur during a chemical reaction [105]. The first work involving DNNs for this issue was presented by Segler et al. [79], published in Nature. They found that Monte Carlo tree search (MCTS) combined with DNNs and symbolic rules can be utilized to perform chemical synthesis effectively. The routes generated by the model were comparable to those reported in the literature in a double-blind AB test, thereby confirming the accuracy of the model [79]. However, it is worth noting that template-based approaches cannot be extended beyond templates, limiting their predictive ability [106].

As for the template-free method, it aims to uncover hidden relationships within the data concerning reaction mechanisms rather than relying on direct matching [105]. For example, by using neural sequence-to-sequence models, Liu et al. [80] proposed the template-free method called seq2seq, to perform the retrosynthetic reaction-prediction tasks. This model was based on an encoder–decoder framework consisting of two recurrent neural networks (RNNs) and was trained on a dataset of 50,000 experimental reactions extracted from the United States’ patent literature, demonstrating comparable performances to the rule-based expert system model [80]. Therefore, ML algorithms have been extensively employed to conduct retrosynthetic analysis and hold the potential to facilitate chemical synthesis.

#### 2.4.2. Forward Reaction Prediction

Contrary to retrosynthesis analysis, forward reaction prediction aims to identify potential molecules that can be synthesized from given reactants and reagents [105]. Given the reactant molecules as input, the ML model analyzes their structural and chemical properties to generate predictions about the resulting products and reaction conditions. For example, Wei et al. [82] introduced a novel reaction fingerprinting approach that utilizes neural networks to predict reaction types. The prediction results of this method on 16 basic reactions of alkyl halides and alkenes indicates that neural networks can contribute to identify key features from the structure of reactant molecules to classify new reaction types [82]. Similarly, Coley et al. [83] proposed a neural network model to predict the main products of chemical reactions by training the data extracted from a collection of 150,000 compounds’ reaction templates in the US patent database. In addition, in practical chemical synthesis reactions, reaction conditions (e.g., solvent and temperature) are critical to maximize the yield of desired products. Based on this, Gao et al. [84] proposed a neural network model to predict the optimal reaction conditions for various types of reactions. This model was trained using a vast dataset of nearly 10 million entries extracted from the Reaxys database and can effectively predict the ideal catalyst, solvent, reagent, and temperature for a given reaction, facilitating the optimization of reaction conditions [84]. Therefore, the utilization of ML-based models can assist in predicting reaction types, accelerating the discovery of new chemical molecules, and identifying optimal reaction conditions, thereby holding great potential in improving the efficiency of chemical synthesis processes.

## 3. Opportunities for Transformer-Based ML Models in Empowering Drug Discovery

**Figure 3:**
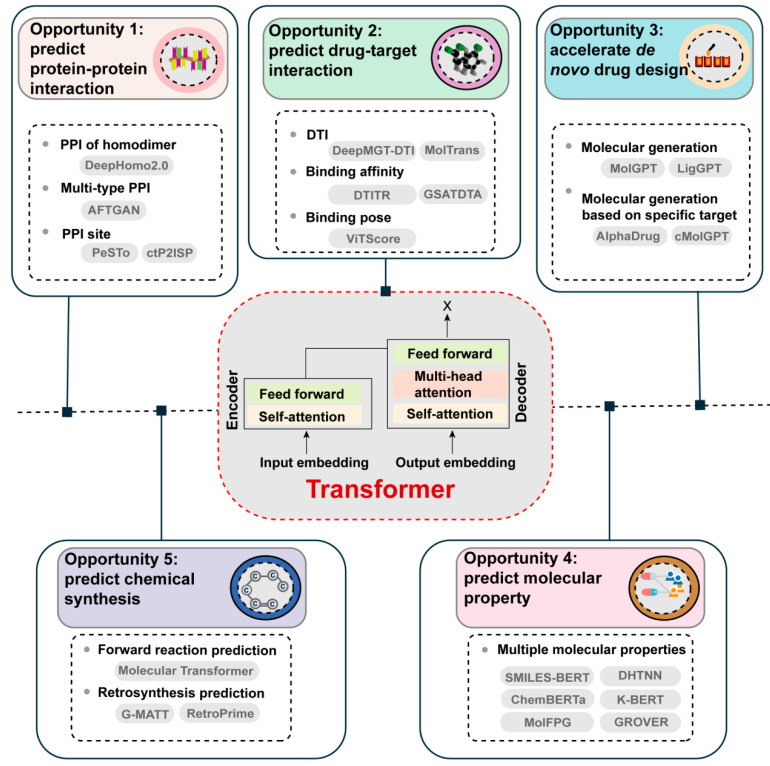
Opportunities for Transformer-based models in empowering drug discovery.

The Transformer model, firstly proposed in the paper ‘Attention is All You Need’ by Vaswani et al., is a highly advanced DL architecture utilizing self-attention mechanisms. As it allows for parallelization and captures long-range dependencies more efficiently than traditional RNN models, the Transformer model has proven to be highly effective in a wide range of tasks and has set new benchmarks in the corresponding fields [10,11]. Given the advantages of the Transformer, it has emerged as a promising future direction of ML in the field of drug discovery (Figure 3).

### 3.1. Opportunity 1: Transformer Models Empowering PPIs Identification

Existing ML-based approaches mainly use CNNs to extract low-dimensional features from protein sequences based on the amino acid composition, while disregarding the long-range relationships within these sequences [107]. Fortunately, transformers can capture the long-distance dependencies in the protein sequences, making them suitable to predict whether and how given proteins interact with each other [108]. For example, by utilizing the advantage of the Transformer model in evolutionary scale modeling-multiple sequence alignment, Lin et al. [109] developed DeepHomo2.0, a DL-based model that predicts PPIs of homodimeric complexes by combining Transformer features, monomer structure information, and direct-coupling analysis. The results showed that DeepHomo2.0 can achieve a high accuracy of over 70% and 60% in terms of experimental monomer structure and predicted monomer structure for the top 10 contacts predicted on the Protein Data Bank (PDB)test set, respectively, which is superior to the DCA-based, protein language model-based and other ML-based methods [109]. Similarly, Kang et al. [110] proposed AFTGAN, a neural network that combines Transformer and GAT frameworks for effective protein information extraction and multi-type PPI prediction. Experimental comparisons validated the superior performance of AFTGAN in accurately predicting the PPIs of unknown proteins. Therefore, given the advantage of the Transformer in extracting protein sequences, it has demonstrated remarkable potential in advancing the prediction of PPIs.

### 3.2. Opportunity 2: Transformer Models Empowering DTIs’ Identification

Despite the remarkable performance improvement of DL models in DTI prediction, the primary challenge lies in the limited representation of drugs in these methods, as they only consider SMILES sequences, SMARTS strings or molecular graphs, failing to capture comprehensive drug representations [107]. It is worth noting that Transformers can be employed either independently or in combination with other AI algorithms to address these problems. For example, DeepMGT-DTI, a DL model that incorporates a Transformer network and multilayer graph information, can effectively capture the structural features of drugs, leading to improved DTI prediction [111]. Experiments have demonstrated that the DeepMGT-DTI can achieve an AUC of 90.24%, an AUPR of 77.11%, an F1 score of 79.31% and an accuracy of 85.15% on the DrugBank dataset. These performance metrics surpassed those previous target sequence structure models, such as Deep DTA and TransformerCPI [111]. Moreover, GSATDTA, a novel triple-channel model based on graph–sequence attention and Transformer, has been developed to predict the drug-target binding affinity with outstanding performance [107]. Therefore, Transformer models have shown promising results for DTIs’ prediction.

### 3.3. Opportunity 3: Transformer Models Empowering De Novo Drug Design

Most existing deep generative models either focus on virtual screening on the available database of compounds by DTI binding-affinity prediction, or unconditionally generate molecules with specific physicochemical and pharmacological properties, which ignore the function of protein targets during the generation process [112]. In contrast, Transformer models have the capability to consider the protein target and achieve target-specific molecular generation. For example, AlphaDrug, a method for protein target-specific *de novo* drug design, has been recently proposed. It utilizes a modified Transformer to optimize the learning of protein information and integrates an efficient MCTS guided by the Transformer’s predictions as well as docking values [112]. Notably, in terms of average docking score, uniqueness, the octanol–water partition coefficient logP, the quantitative estimate of drug-likeness (QED), synthetic accessibility (SA) and Natural products-likeness (NP-likeness) criteria, AlphaDrug is superior to other methods (such as LiGANN, SBMolGen and SBDD-3D) [112]. In addition, the GPT model is a powerful language generation model that can be fine-tuned for specific tasks after pre-training on large amounts of text data [113]. It has been successfully applied to accelerate molecular generation for specific targets in the field of drug discovery. For example, cMolGPT, a GPT-inspired model, is a useful tool for target-specific *de novo* molecular generation. The chemical space of the compounds generated by cMolGPT closely matches with that of real target-specific ones [114].

### 3.4. Opportunity 4: Transformer Models Empowering Molecular Property Prediction

Despite the widespread application of ML-based models, the shortage of labeled data continues to be a significant challenge in efficient molecular property predictions [10,115]. To address this, researchers are exploring the use of unlabeled data and leveraging transformer-based self-supervised learning (e.g., BERT) to improve predictions on small-scale labeled data [116]. Currently, several BERT-based pre-training methods for molecular property prediction have been proposed [10,117]. For example, a novel pre-training method, known as K-BERT, was developed to extract chemical information from SMILES similar to chemists for molecular property prediction in drug discovery [118]. The K-BERT model exhibited superior performance in 8 out of 15 tasks, thus reflecting the efficacy and benefits of the proposed pre-training approach in drug discovery. Specifically, K-BERT had an average AUC score of 0.806, outperforming other competing methods (e.g., XGBoost-MACCS, XGBoost-ECFP4, HRGCN+ and Attentive FP) [118]. Moreover, Wang et al. [119] proposed a two-stage (pre-training and fine-tuning) model called SMILES-BERT that could use both unlabeled data and labeled data to improve molecular property prediction. Compared with a range of state-of-the-art approaches (e.g., CircularFP, NeuralFP, Seq2seqFP, Seq3seqFP), it exhibited superior performance on three different datasets (the LogP dataset, PM2 dataset and PCBA-686978 dataset) with accuracies of 0.9154, 0.7589, and 0.8784, respectively [119]. Therefore, these Transformer-based predictors are essential tools for molecular property prediction, contributing to the efficient screening of potential drug candidates.

### 3.5. Opportunity 5: Transformer Models Empowering Chemical Synthesis

Previous sequence-based approaches commonly employed RNNs for both the encoder and decoder, with a single-head attention layer connecting them. These models treated reactants and reagents separately in the input by utilizing atom mapping, which limits the interpretability of the model [120]. Fortunately, Transformer-powered models have shown potential to accelerate chemical synthesis. One notable example is the effectiveness of the multi-head attention Molecular Transformer model in predicting chemical reactions and reaction conditions [120,121]. In addition, inspired by the success of the Molecular Transformer for forward reaction prediction, Schwaller et al. [122] proposed an enhanced Molecular Transformer architecture coupled with a hyper-graph exploration algorithm for automated retrosynthetic pathway prediction. This approach surpasses previous ML-based methods by not only predicting reactants but also identifying reagents for each retrosynthetic step, thereby significantly raising the complexity of the prediction task.

## 4. Challenges of ML-Based Models in Drug Discovery

**Figure 4:**
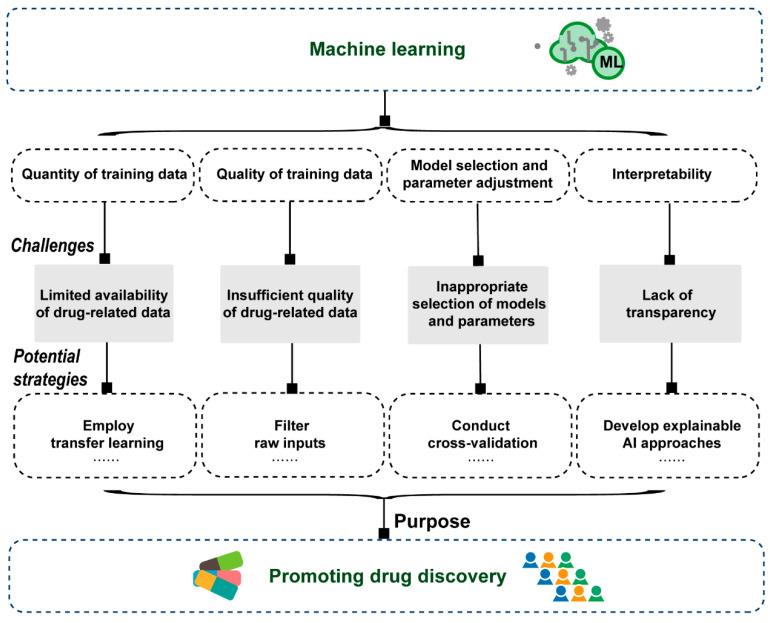
Challenges of machine learning-based models in drug discovery.

Given the remarkable advantages in identifying and extracting features from high-dimensional and complex big data, ML-based models have made significant progress in multiple stages of drug discovery [99]. However, there remain several challenges that have yet to be effectively resolved (Figure 4).

First, the effectiveness of ML algorithms heavily relies on the quantity of training data, and typically, a larger dataset tends to yield a more accurate model [96]. When the amount of data is inadequate, it can significantly impact the performance and reliability of ML models, potentially resulting in the risk of overfitting [123]. Indeed, the limited availability of data, especially labeled data, poses a significant challenge to the progress of ML-driven drug discovery. One potential approach to address this issue is employing transfer learning algorithms, where knowledge acquired from one task can be effectively applied to another task [124,125,126]. Additionally, in light of the challenges associated with acquiring extensive labeled datasets in drug discovery, there is a growing trend for the effectiveness of concentrating efforts on smaller, carefully curated datasets. This shift highlights the significance of extracting meaningful insights from limited yet relevant data, thereby enhancing the precision and applicability of ML models in the complex landscape of drug discovery.

Second, the quality of the data is also crucial in determining the prediction performance of ML models. The experimental drug-related data collected in public databases frequently originates from varying biological assays, conditions, or methods, leading to disparate results when different measurement techniques are employed for a specific compound, thereby hindering direct comparisons. Hence, the strategies for filtering raw inputs with noise, outliers, or irrelevant information and automating data entry may be helpful to achieve reliable and accurate ML models for drug discovery. For example, during the data processing phase, noise reduction and outlier detection algorithms, such as *Z*-scores, box plots or iterative deletion, can be applied to identify and purge outliers from the data, enhancing its quality for ML model prediction. In addition, researchers can use cross-validation experiments to assess the generalization ability of the models, ensuring that they perform well not only on specific datasets but also on new, unseen data.

Third, due to the abundance of ML model architectures and the constant emergence of new ones, it becomes challenging to choose the most suitable models that meet specific research task requirements in the field of drug discovery [99]. Generally, the model selection involves evaluating various options and considering factors such as the complexity of the problem, available data, and computational resources. Furthermore, once the model architecture is selected, the next step is to fine-tune its parameters to optimize the model’s performance. Although hyperparameter optimization tools have been proposed to automate the process of tuning substantial parameters in ML models, the entire system process is also relatively complicated, which may bring certain difficulties to the application of researchers [99,127]. In addition, the setting of hyper-parameters usually requires human intervention, which may lead to their incomplete or inaccurate selection. Accordingly, cross-validation is commonly used in variable selection and model parameter tuning to evaluate the performances of various ML methods [128]. Moreover, establishing clear performance metrics at the outset, such as accuracy, precision, recall, F1 score, AUC and AUPR can help in objectively evaluating the suitability of different models depending on the nature of the problem.

Fourth, unlike traditional models where the reasoning and decision-making process can be easily understood, ML models, particularly DL models, operate using complex mathematical algorithms and layers of interconnected neurons, making it challenging to interpret their inner workings. The lack of transparency and interpretability pose difficulties for ML models in explaining the observed phenomena and understanding the underlying biological mechanisms. Hence, the ML models are often referred to as “black boxes” [99]. For this issue, employing visualization tools such as Activation Maximization [129], Local Interpretable Model-agnostic Explanations (LIME) [130] and SHapley Additive exPlanations (SHAP) [131] can help in understanding the model’s decision-making process by providing insights into which features are most influential. In the future, a continuous requirement is to develop robust models with high interpretability.

Therefore, a tremendous amount of work has been done to incorporate ML tools to expedite the drug discovery cycle, but further advancement and improvement of these tools is needed before the full potential of ML in drug discovery can be realized.

## 5. Concluding Remarks

The research and development of new drugs can contribute to meet the human demand for treating diseases and provide more effective, safer, and more convenient treatment options. Compared with the traditional strategies of drug discovery, ML-based approaches have the potential to reduce time and costs, improve safety, and bridge the gap between drug discovery and drug effectiveness, making them increasingly favored by the pharmaceutical industry and academia. In particular, the introduction of chatGPT has sparked researchers’ growing interest and exploration in leveraging the Transformer model’s NLP capabilities, particularly its self-attention mechanisms, to accelerate multiple stages of the drug discovery process, thereby opening up new opportunities for advancements.

However, the current challenges in ML-based models can result in generating false positives or false negatives, potentially leading to incorrect predictions and resource waste. Further in vitro and in vivo experiments as well as clinical trials are needed to fully demonstrate the practicability of ML-based drug discovery and obtain more reliable and accurate results. Therefore, future research should focus on improving data quality, enhancing the interpretability of ML algorithms, and integrating them with human professional knowledge to increase the efficacy of drug discovery.

## Figures and Tables

**Table 1:** ML-based software/model used for drug discovery.

Name	Algorithm	Specific Function	PMID
**Prediction of the target protein structure**			
TrRosetta server	DNN	Predict 3D structures of proteins	[13]
AlphaFold	DNN	Predict 3D structures of proteins	[14]
ComplexQA	GNN	Predict protein complex structure	[15]
ProteinBERT	Transformer	Predict secondary structure	[16]
ESMfold	Transformer	Predict structure of proteins	[17]
**Predicting protein–protein interactions**			
IntPred	RF	Predict PPI interface sites	[18]
eFindSite	SVM; NBC	Predict PPI interfaces	[19]
DELPHI	RNN; CNN	Predict PPI sites	[20]
PPISP-XGBoost	XGBoost	Predict PPI sites	[21]
HN-PPISP	CNN	Predict PPI sites	[22]
TAGPPI	GCN	Predict PPIs	[23]
Struct2Graph	GAT	Predict PPIs	[24]
DeepFE-PPI	DNN	Predict PPIs	[25]
SGPPI	GCN	Predict PPIs	[26]
DeepPPI	DNN	Predict PPIs	[27]
DL-PPI	GNN	Predict PPIs	[28]
DeepSG2PPI	CNN	Predict PPIs	[29]
MaTPIP	Transformer; CNN	Predict PPIs	[30]
ProtInteract	Autoencoder; CNN	Predict PPIs	[31]
**Predicting drug–target interactions**			
DeepC-SeqSite	CNN	Predict DTI binding sites	[32]
DeepSurf	CNN; ResNet	Predict DTI binding sites	[33]
PrankWeb	RF	Predict DTI binding sites	[34]
PUResNet	ResNet	Predict DTI binding sites	[35]
AGAT-PPIS	GNN	Predict DTI binding sites	[36]
DeepDTA	CNN	Predict DTI binding affinity	[37]
SimBoost	GBM	Predict DTI binding affinity	[38]
DEELIG	CNN	Predict DTI binding affinity	[39]
DeepDTAF	CNN	Predict DTI binding affinity	[8]
GraphDelta	CNN	Predict DTI binding affinity	[40]
PotentialNet	CNN	Predict DTI binding affinity	[41]
DeepAffinity	RNN, CNN	Predict DTI binding affinity	[9]
TeM-DTBA	CNN	Predict DTI binding affinity	[42]
Wang et al.’s method	RL	Predict DTI binding pose	[43]
Nguyen et al.’s method	RF; CNN	Predict DTI binding pose	[44]
AMMVF-DTI	GAT; NTN	Predict drug–target interactions	[45]
***De novo* drug design**			
ReLeaSE	RNN; RL	Conduct *de novo* drug design	[46]
ChemVAE	CNN; GRU	Conduct de novo drug design	[47]
MolRNN	RNN	Conduct multi-objective *de novo* drug design	[48]
PaccMann(RL)	VAE	Generate compounds with anti-cancer drug properties	[49]
druGAN	AAE	Conduct *de novo* drug design	[50]
SCScore	CNN	Evaluate the molecular accessibility	[51]
UnCorrupt SMILES	Transformer	Conduct *de novo* drug design	[52]
PETrans	Transfer learning	Conduct *de novo* drug design	[53]
FSM-DDTR	Transformer	Conduct *de novo* drug design	[54]
DNMG	GAN	Conduct *de novo* drug design	[55]
MedGAN	GAN	Design novel molecule	[56]
**Prediction of the physicochemical properties**			
Panapitiya et al.’s method	GNN	Predict aqueous solubility	[57]
SolTranNet	Transformer	Predict aqueous solubility	[58]
Zang et al.’s method	SVM	Predict multiple physicochemical properties	[59]
**Prediction of the ADME/T properties**			
ADMETboost	XGBoost	Predict ADME/T properties	[60]
vNN	k-NN	Predict ADME/T properties	[61]
Interpretable-ADMET	CNN; GAT	Predict ADME/T properties	[62]
XGraphBoost	GNN	Predict ADME/T properties	[63]
DeepTox	DNN	Predict toxicity of compounds	[64]
Li et al.’s method	DNN	Predict human Cytochrome P450 inhibition	[65]
LightBBB	LightGBM	Predict blood–brain barrier	[66]
Deep-B3	CNN	Predict blood–brain barrier	[67]
PredPS	GNN	Predict stability of compounds in human plasma	[68]
Khaouane et al.’s method	CNN	Predict plasma protein binding	[69]
**Application of AI in drug repurposing**			
deepDTnet	Autoencoder	Predict new targets of known drugs	[70]
NeoDTI	GCN	Predict new targets of known drugs	[71]
DTINet	Network diffusion algorithm and the dimensionality reduction	Predict new targets of known drugs	[72]
MBiRW	Birandom walk algorithm	Predict new indications of known drugs	[73]
GDRnet	GNN	Predict new indications of known drugs	[74]
deepDR	VAE	Predict new indications of known drugs	[75]
GIPAE	VAE	Predict new indications of known drugs	[76]
DrugRep-HeSiaGraph	Heterogeneous siamese neural network	Predict new indications of known drugs	[77]
iEdgeDTA	GCNN	Predict DTI binding affinity	[78]
**Retrosynthesis prediction**			
Segler et al.’s method	MCTS, DNN	Predict retrosynthetic analysis	[79]
Liu et al.’s method	RNN	Predict retrosynthetic analysis	[80]
RAscore	RF	Predict retrosynthetic accessibility score	[81]
**Reaction prediction**			
Wei et al.’s method	Neural network	Predict reaction classes	[82]
Coley et al.’s method	Neural network	Predict products of chemical reactions	[83]
Gao et al.’s method	Neural network	Predict optimal reaction conditions	[84]
Marcou et al.’s method	RF	Evaluate reaction feasibility	[85]

Note: DNN, deep neural network; RNN, recurrent neural network; RF, random forest; CNN, convolutional neural network; XGBoost, eXtreme gradient boosting; GCN, graph convolutional network; GAT, graph attention network; SVM, support vector machine; NBC, naïve Bayes classifier; ResNet, residual network; GBM, gradient boosting machines; RL, reinforcement learning; GRU, gated recurrent unit; VAE, variational autoencoder; AAE, adaptive adversarial autoencoder; GNN, graph neural networks; k-NN, k-nearest neighbor; LightGBM, light gradient boosting machine; MCTS, Monte Carlo tree search, NTN, neural tensor network; GAN, generative adversarial network; GCNN, graph convolutional neural network.

## Data Availability

Not applicable.

## Abbreviations

AI, artificial intelligence; ML, machine learning; DL, deep learning; ANN, artificial neural network; NLP, natural language processing; DTI, drug–target interaction; 3D, three-dimensional; DNN, deep neural network; PPI, protein–protein interaction; GAT, graph attention network; CNN, convolutional neural network; VAE, variational auto-encoder; AAE, adversarial auto-encoder; RNN, recurrent neural network; RF, random forest; XGBoost, eXtreme gradient boosting; GCN, graph convolutional network; SVM, support vector machine; NBC, naïve Bayes classifier; NTN, neural tensor network; GAN, generative adversarial network; GCNN, graph convolutional neural network; ResNet, residual network; GBM, gradient boosting machines; RL, reinforcement learning; GRU, gated recurrent unit; GNN, graph neural networks; k-NN, k-nearest neighbor; LightGBM, light gradient boosting machine; MCTS, Monte Carlo tree search, NTN, neural tensor network; GAN, generative adversarial network; GCNN, graph convolutional neural network; ADME/T, absorption, distribution, metabolism, and excretion and toxicity; BBB, blood–brain barrier; PPB, plasma protein binding, CYP2D6, cytochrome P450 2D6; XGBoost, extreme gradient boosting; CYP450, cytochrome P450;MCTS, Monte Carlo Tree Search; GPT, Generative Pre-Training Transformer; BERT, bidirectional encoder representations from transformers; SC2, Challenge to Predict Aqueous Solubility; MCC, Matthews’ Correlation Coefficient; AUC, area under the curve; AUPR, area under precision-recall; PDB, Protein Data Bank; QED, quantitative estimate of drug-likeness; SA, synthetic accessibility;NP, natural products; LIME, Local Interpretable Model-agnostic Explanations; SHAP, SHapley Additive exPlanations.
